# Time doesn’t heal all: PTSD symptoms exacerbate the relationship between age and pain intensity

**DOI:** 10.3389/fpsyt.2023.1221762

**Published:** 2023-07-27

**Authors:** Victoria O’Connor, Jared A. Rowland, Jennifer C. Naylor, Anna T. Magnante, Katherine M. Craig, Holly M. Miskey, VA Mid-Atlantic MIRECC Workgroup, Jean C. Beckham, Sarah L. Martindale

**Affiliations:** ^1^W. G. (Bill) Hefner VA Healthcare System, Salisbury, NC, United States; ^2^Veterans Integrated Service Networks (VISN)-6 Mid-Atlantic Mental Illness, Research Education and Clinical Center (MIRECC), Durham, NC, United States; ^3^Wake Forest School of Medicine, Winston-Salem, NC, United States; ^4^Durham Veterans Affairs Health Care System, Durham, NC, United States; ^5^Department of Psychiatry and Behavioral Sciences, Duke University School of Medicine, Durham, NC, United States

**Keywords:** aging, posttraumatic stress disorder, veterans, pain, pain intensity

## Abstract

**Objective:**

Posttraumatic stress disorder (PTSD) symptoms and pain interfere with daily functioning and quality of life for many combat Veterans. As individuals age, pain symptoms tend to increase whereas PTSD symptoms tend to decrease. PTSD symptoms exacerbate pain, but the nature of this relationship across the aging process is unclear. The purpose of this study was to determine how PTSD symptoms affect the association between age and pain intensity.

**Methods:**

Participants in this cross-sectional study included 450 Veterans (80% male) who served after September 11, 2001. PTSD and pain intensity ratings were assessed by the PTSD Checklist for DSM-5 (PCL-5) and the Brief Pain Inventory (BPI), respectively. Hierarchical multiple linear regression evaluated main and interaction effects between age, PTSD symptoms, and pain intensity.

**Results:**

Age (*B* = 0.04, *p* < 0.001) and PTSD symptoms (*B* = 0.05, *p* < 0.001) were positively associated with pain intensity. Age and PTSD symptoms were inversely correlated (*r* = −0.16, *p* < 0.001). PTSD symptoms exacerbated the relationship between age and pain intensity (Δ*R^2^* = 0.01, *p* = 0.036). Specifically, when greater PTSD symptoms were reported at older ages, pain intensity was significantly higher.

**Conclusion:**

Results of these analyses suggests that age is important when considering the effects of PTSD symptoms on pain intensity ratings. Specifically, pain intensity ratings are higher in older Veterans with PTSD symptoms. These findings underscore the importance for clinical providers to evaluate trauma history and PTSD symptoms in older Veterans reporting pain symptoms.

## Introduction

The relationship between psychiatric distress and pain is complex and dynamic. Greater posttraumatic stress symptom burden contributes to increased pain intensity (1, 2), however both posttraumatic stress and pain are differentially associated with age. Specifically, posttraumatic stress symptoms decrease with age (3, 4), whereas pain intensity tends to increase with age (5, 6). Although PTSD symptom distress may independently affect pain intensity, there is little evidence about how PTSD symptom distress affects pain severity across advancing age. The purpose of this study was to determine if and how posttraumatic stress disorder (PTSD) symptoms affected the relationship between age and pain.

Pain is a common presenting concern for older adults seeking medical treatment (7) and is associated with decreased quality of life (8), greater disability (9), increased risk for suicide (10), and significant health care costs (11). Most research examining pain has focused on chronic pain (i.e., pain lasting for >3 months), which is the most commonly reported health concern of post-9/11 military Veterans (12, 13). Although work with chronic pain is important, evaluating general pain intensity across any duration is an equally vital consideration given the increased likelihood of events (e.g., injuries related to falls, chronic conditions such as arthritis, surgeries) that can cause pain and are associated with advancing age (14, 15). Approximately 66% of Veterans report pain, with 9% reporting severe pain (12). This is likely related the increased risk for physical injury during combat or trainings as well as exposure to physical and environmental stressors that occur during military service. Congruently, pain is associated with a number of adjacent concerns, including mental health conditions, that contribute to increased health care burden (8).

PTSD similarly contributes to the substantial health care burden for many military Veterans. PTSD is the most prevalent mental health diagnosis and third most common service-connected disability among Veterans (16). Contrary to the typical trajectory of pain, the severity and prevalence of PTSD symptoms typically decline with age (3, 4), for which several potential contributing factors exist. Increased time since the trauma potentially allows individuals time to seek treatment, process and draw meaning from their traumatic experience, leading to reduced avoidance behaviors (17–19). Additionally, older adults generally endorse greater meaning in life, spirituality, and resilience, each of which have been demonstrated to buffer PTSD symptoms (20–22). Early mortality, associated with the high prevalence of comorbid chronic health conditions, may also contribute to the perception of decreased PTSD prevalence in older adults (23, 24).

Although both pain and PTSD symptoms can negatively affect quality of life, they can be particularly impairing when they co-occur. Chronic pain and PTSD have a reciprocal relationship, negatively and continuously affecting symptoms of the other, while cumulatively exacerbating negative functional outcomes (25–27). This reciprocal relationship falls under the biopsychosocial model of chronic pain that recognizes the dynamic interactions between biological, psychological, and social factors in the pain experience (28). There are numerous theories proposing mechanisms that explain the reciprocal relationship between PTSD and pain, including perceptual alterations of pain (e.g., anxiety sensitivity), coping, and cognitive changes [for a detailed review, please see (29)]. Investigations of pain and PTSD often evaluate the magnitude and directionality of the relationship (30–32). For example, Veterans with PTSD report greater pain intensity than Veterans without PTSD (26, 33). Additionally, the relationship between PTSD and chronic pain has been shown to be fairly consistent over a 3 year period among young adult military personnel (34). However, little is known about the effect of age on the relationship between PTSD and pain. A recent meta-analysis reported that the relationship between PTSD and pain remains important at older ages in civilian samples (35). Considering the high prevalence of pain and PTSD in Veteran samples, this is an especially important question to evaluate within this population.

In summary, PTSD symptom distress may be an important factor to consider in the relationship between age and pain. Pain and PTSD symptoms are differentially associated with age; pain intensity increases with age, whereas PTSD symptoms decline with age. Given that PTSD is often associated with increased pain, it is likely that PTSD symptoms affect pain intensity ratings at older ages. The purpose of the present analysis was to understand how PTSD symptom severity and age interact to affect pain intensity. It was hypothesized that age would be positively associated with pain intensity and negatively correlated with PTSD symptom severity. Second, it was hypothesized that PTSD symptom severity would exacerbate the association between age and pain intensity. These preliminary analyses inform whether addressing PTSD symptoms represent an additional avenue of treatment to help alleviate experience of pain, specifically as Veterans age and the incidence of pain increases.

## Method

Data were collected as part of the VISN 6 Mental Illness, Research, Education and Clinical Center’s (MIRECC) Study of Post-Deployment Mental Health Longitudinal Follow Up (PDMH-L). PDMH-L is the longitudinal arm of the baseline multisite PDMH study (36). The purpose of the PDMH studies is to better understand the effect of deployment on the physical and mental health of service members and Veterans. These studies gather clinical interviews, biological samples, questionnaires, physical measurements, and data from the electronic health record. The study was approved by the respective IRBs at each site. Participants were reimbursed for their time and travel.

### Participants

The inclusion criterion for the baseline PDMH study was service in the US military since 09/11/2001. The baseline PDMH visit had no exclusion criteria. Data for the present analyses were cross-sectional and collected during the first follow-up PDMH-L visit and included the first 490 participants enrolled between 2018 and 2022. Because this is a longitudinal protocol, enrollment is ongoing. Participants from the current study were excluded from these analyses if they scored above threshold (>23) on the Structed Inventory for Malingered Symptoms (SIMS; *n* = 40) (37, 38). There was no missing data after excluding participants for symptom validity. The final sample size for analyses was *N* = 450.

### Measures

The PTSD Checklist for DSM-5 (PCL-5) is a 20-item self-report measure of PTSD symptom burden (39). Participants rate each item based on how much the symptom has bothered them over the past month. Items are rated from 0 (not at all) to 4 (extremely), with total scores ranging from 0 to 80. The PCL-5 has demonstrated good reliability (*α* = 0.96) in samples of military Veterans (40).

The short form of the Brief Pain Inventory (BPI) is a nine item self-report measure of pain experienced over the past 24 h (41). Participants rated their pain across four time periods: worst, least, average, and current pain. Items are rated on a scale from 0 (no pain) to 10 (pain as bad as you can imagine). Items are summed to create scores for pain severity and pain interference. The average pain severity scale, comprised of four items, was used in the present analyses to assess pain intensity. The *interference in general activity* item was used for the exploratory analysis assessing pain interference. The average pain intensity scale of the BPI has demonstrated good reliability (*α* = 0.85) in previous samples (42).

The Structured Clinical Interview for the DSM-IV-TR Disorders [SCID-IV; (43)] assessed the presence of current PTSD diagnosis. The SCID-IV is a structured clinical interview facilitated by trained research assistants and providers.

#### Covariates

Participants completed several measures as part of the PDMH-L. Gender, TBI status, combat experiences, deployment status, time since deployment, medical conditions, pain medications, and education were investigated as potential covariates, theoretically determined based on prior literature of relationship to pain. Combat exposure was measured using Section D of the Deployment Risk and Resilience Inventory, second edition [DRRI-2; (44, 45)]. Health and medical conditions were identified using the National Vietnam Veterans Readjustment Study Self-Reported Medical Questionnaire [NVVRS; (46)]. Traumatic brain injury (TBI) was evaluated using the Mid-Atlantic MIRECC Assessment of Traumatic Brain Injury [MMA-TBI; (44)].

### Data analysis

Data were analyzed with SAS Enterprise Guide 8.3 (SAS Institute, Inc., Cary, NC). Continuous variables of interest were normally distributed. Correlations for continuous data and independent samples *t*-tests for dichotomous data were conducted between potential covariates of interest (i.e., gender, years of education, race, ethnicity, combat exposure, any TBI history, pain medication, health conditions) and pain outcomes to determine appropriateness of inclusion in analyses. Four self-reported conditions on the NVVRS (i.e., asthma; diabetes; trouble with head, back or spine; stiffness) were significantly associated with pain intensity. Combat exposure (DRRI-2-D) was significantly correlated with pain intensity. The four medical conditions and combat exposure were included as covariates across all analyses. TBI, education, race, ethnicity, pain medications, and gender were not significantly associated with pain intensity and were not included as covariates.

Hierarchical regression analysis, adjusting for covariates across all steps, evaluated the main effects of age and PTSD symptoms on pain intensity. The first step of the model evaluated the main effects of age and PTSD symptoms on pain intensity in addition to covariates. The second step of the model included the interaction term between age and PTSD symptoms. All data are cross-sectional and do not infer causality. The interaction effect was calculated using the PROCESS macro v.4.2 (47) and Johnson-Neyman analysis (48) was used to probe significant interaction effects. Presented 95% confidence intervals are bootstrapped with 10,000 resamples. The variance inflation factor ranged from 1.01 to 1.21 across models, indicating low collinearity.

## Results

Participants were 450 post-9/11 Veterans (80% male) between the ages of 33 and 78 (Table 1), normally distributed within descriptive categories of younger adults (16% ages 18–39), middle-aged adults (64% ages 40–59), and older adults (19% ages 60+). Most participants identified as White (49%) or Black (48%) with some college education (*M_years_* = 14.36). The majority of participants (95%) reported at least one combat deployment during their military service. The sample typically endorsed PTSD symptoms considered to be well below threshold [i.e., > 33; (40)] for diagnosis (*M* = 21.97; SD = 19.29). Approximately one fifth of the sample met full diagnostic criteria for a current PTSD diagnosis (*n* = 88; 19.64%) according to the SCID-IV (43). Most participants endorsed chronic pain (*n* = 373, 84.19%) evaluated as endorsing pain on item 10 of the BPI persisting greater than 3 months.

**Table 1:** Sample demographics (*N* = 450).

Demographic	*M* (SD), range or *n* (%)
Age	50.71 (9.75), 33–78
Education^a^	14.36 (2.05), 10–22
Combat deployment	426 (94.67%)
Number of deployments	1.47 (1.92), 0–11
Years since most recent deployment^b^	15.56 (5.56), 1–52
Gender	
Male	363 (80.67%)
Female	87 (19.33%)
Race^c^	
White/Caucasian	219 (49.10%)
Black/African American	217 (48.65%)
Other	15 (3.36%)
Branch of Service	
Air force	30 (6.87%)
Army	278 (63.62%)
Marine corps	44 (10%)
Navy	60 (13.73%)
Coast guard	1 (0.23%)
Unknown/Did not report	37 (5.55%)
Traumatic brain injury history	152 (33.56%)
Current PTSD Diagnosis	88 (19.64%)
Chronic Pain	373 (84.19%)

a Missing years of education (*n* = 20).

b Only calculated among participants who deployed.

c Categories not mutually exclusive.

Number of deployments includes contract deployments to warzones in support of the wars in Iraq and Afghanistan. Race was collapsed due to small numbers in categories; “Other” includes Native America/Alaska Native, Asian, Other, and Refused. Branch of Service indicates most recent branch. Branches of service include Reserve and Guard. PTSD Diagnosis was determined using the Structured Clinical Interview for DSM-IV Disorders, which all participants completed; SCID-IV diagnoses were not included in analyses. Chronic Pain > 3 months.

As presented in Table 2, pain intensity was significantly, positively correlated with age (*r* = 0.11, *p* = 0.021) and PTSD symptoms (*r* = 0.36, *p* < 0.001). PTSD symptoms were significantly, negatively correlated with age (*r* = −0.17, *p* < 0.001). There were no significant sex differences on pain intensity (*p* = 0.073), pain interference (*p* = 0.510), or PCL-5 scores (*p* = 0.832). However, men (*M* = 51.25) were reliably older than women (*M* = 48.52) in this sample, *t* (448) = 2.35, *p* = 0.019.

**Table 2:** Relationships between PTSD symptoms, age, and pain intensity.

Measure		Correlations (*r*)			Measure characteristics		
		1	2	3	*M*	SD	Range
1	Pain intensity	–			3.64	2.40	0–10
2	Age	0.108^*^	–		50.71	9.75	33–78
3	PTSD symptoms	0.364^**^	−0.166^**^	–	21.97	19.29	0–78
4	Pain interference	0.760^**^	0.015	0.498^**^	3.97	3.17	0–10

^*^< 0.05. ^**^< 0.01; Pain Intensity and Pain Interference are derived from the Brief Pain Inventory Numerical Severity Rating; PTSD Symptoms = PTSD Checklist for DSM-5.

As presented in Table 3, the omnibus model was statistically significant, *R*^2^ = 0.20, *p* < 0.001. In Step 1, there were significant main effects of age, *B* = 0.04, *p* < 0.001, CI [0.02, 0.06], and posttraumatic stress symptoms, *B* = 0.05, *p* < 0.001, CI[0.03, 0.06] on pain intensity beyond covariates.

**Table 3:** Hierarchical regression evaluating main and interaction effects of age and PTSD symptoms on pain intensity.

Model (Outcome: Pain severity)	Omnibus model			Parameter estimates					
	*R* ^2^	*p*	Δ*R*^2^ sig	*B*	SE	*t*	*p*	LLCI	ULCI
Step 1	0.20	< 0.001	< 0.001						
Age				0.042	0.01	3.93	< 0.001	0.021	0.064
PTSD Symptoms				0.047	0.01	8.18	< 0.001	0.036	0.059
Step 2	0.22	< 0.001	< 0.001						
Age				0.001	0.02	0.58	0.564	−0.023	0.041
PTSD Symptoms				−0.003	0.03	−0.97	0.330	−0.084	0.029
Age^*^ PTSD Symptoms				0.001	0.01	2.09	0.036	0.001	0.003

The reported hierarchical regression adjusts for covariates (i.e., combat exposure and medical conditions) across all steps.

**Figure 1:**
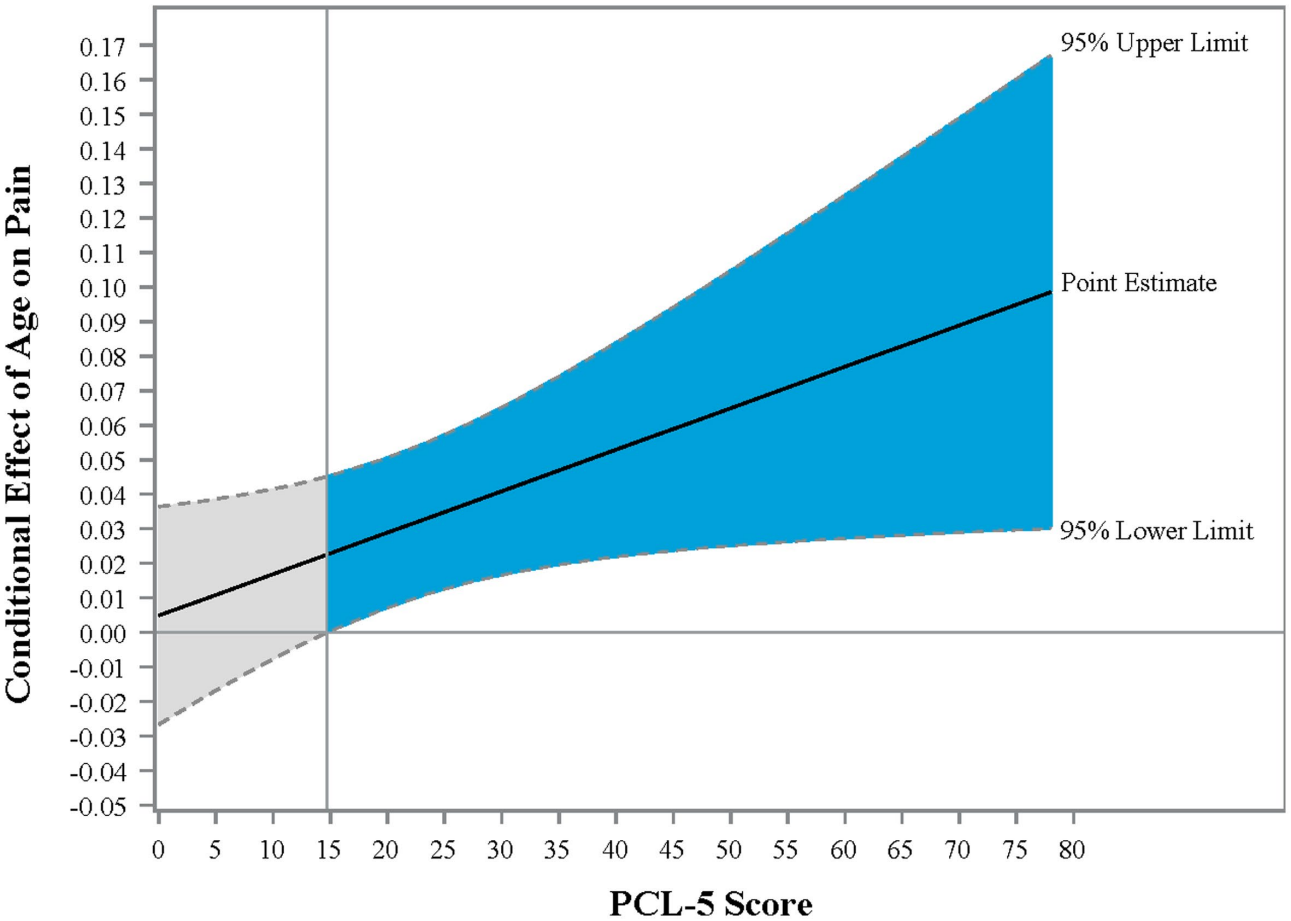
Johnson-Neyman graph illustrating the conditional effect of PTSD on the relationship between age and pain intensity. Johnson-Neyman analysis provides the point of the moderator at which the effect of the independent variable on the dependent variable is significant. In the present sample, the effect of age on pain is significant when PCL-5 scores were above 14.72. Blue shading reflects the region of significance.

**Figure 2:**
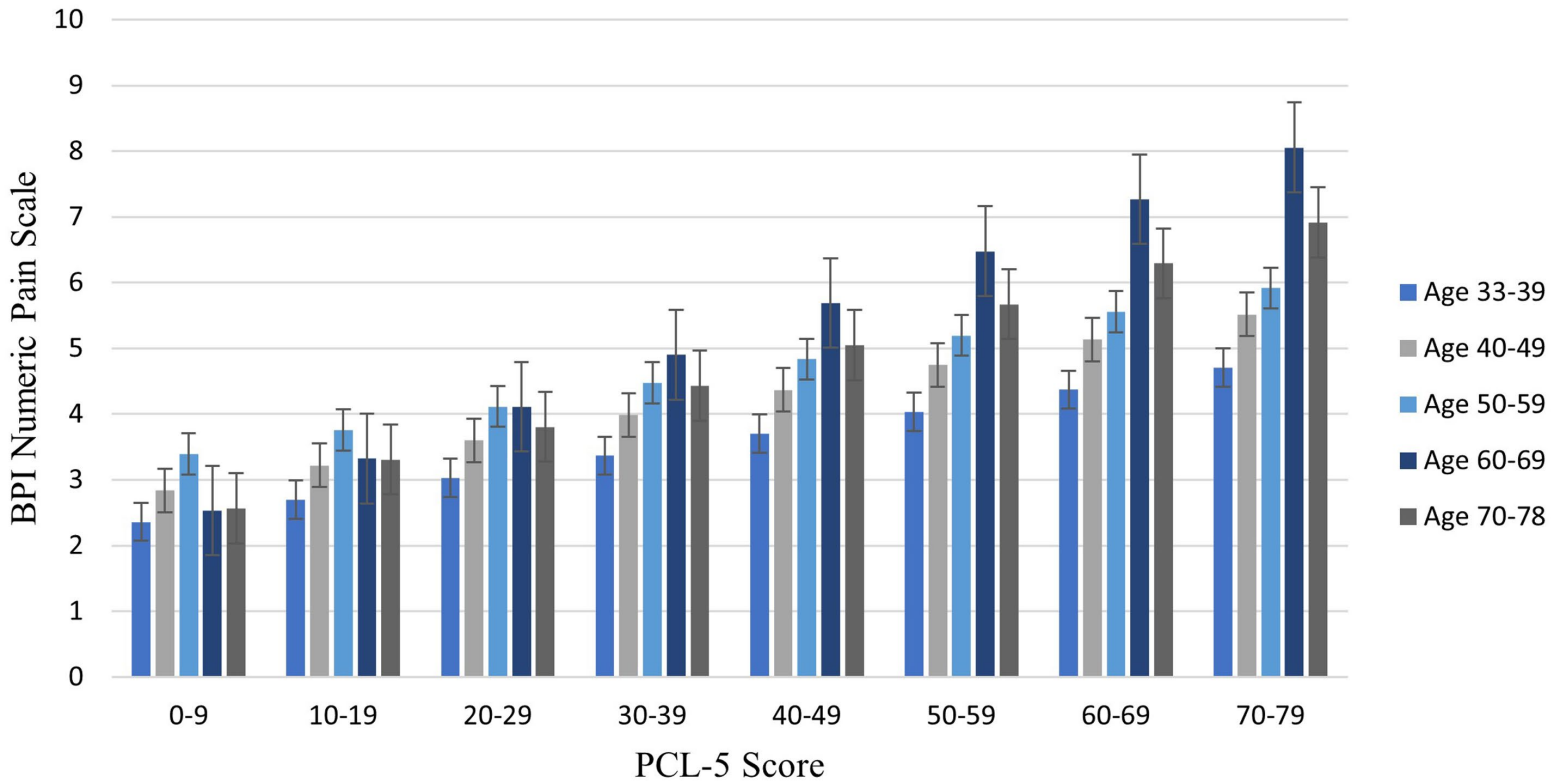
Bar graph illustrating average pain intensity at different PTSD symptom severity scores by decade of life. This figure groups age by decade and PCL scores in 10-point increments for illustrative purposes only; continuous age and PCL-5 scores were utilized in analyses. Sample size for age groups: 30–39 (*n* = 74, 16.44%); 40–49 (*n* = 127, 28.22%); 50–59 (*n* = 164, 36.44%); 60–69 (*n* = 71, 15.78%); 70–79 (*n* = 14, 3.11%). BPI = Brief Pain Inventory. PCL-5 = PTSD Checklist.

The interaction effect between age and posttraumatic stress symptoms in Step 2 was significant, Δ*R*^2^ = 0.01; *F* (1,439) = 4.39, *p* = 0.036, CI [0.001, 0.002], demonstrating that the effect of age on pain was enhanced as PTSD symptom severity increased. Johnson-Neyman analysis indicated that the effect of age on pain severity was significant when PCL-5 scores were above 14.72 (Figure 1). Of note, age and PTSD symptoms were used as continuous variables for statistical analyses and were grouped only for illustration purposes in the figure. Pain scores were exacerbated with increasing PCL-5 scores (Figure 2). These effects were strongest among the oldest participants.

### Exploratory analyses

Because pain interference is also a construct of interest, parallel analyses examined pain interference (*M* = 3.97, SD = 3.17) as an outcome. The omnibus model for step 1 was statistically significant, *R*^2^ = 0.22, *p* < 0.001. There was a significant main effect for posttraumatic stress symptoms on pain interference, *B* = 0.06, *t* = 8.22, *p* < 0.001, but not age, *B* = 0.01, *t* = 0.93, *p* = 0.355. The interaction effect between age and PCL-5 total score was also not significant, *B* = 0.001, Δ*R*^2^ = 0.005, *p* = 0.110.

## Discussion

The results of the present study demonstrate that the interaction between PTSD symptoms and age is important for understanding pain intensity in Veterans. As expected, pain intensity increased with age and PTSD symptoms, and PTSD symptoms declined with age (Table 2). However, the effect of age on pain was exacerbated as self-reported PTSD symptoms increased. Specifically, scores of 15 or greater on the PCL-5 were associated with increasing effects of age on pain intensity. A score of 15 is below the threshold (> 33) generally considered clinically significant (40); however, these findings emphasize that even low levels of self-reported PTSD symptoms can begin to affect health.

These results also demonstrate that PTSD symptoms contribute to higher pain ratings in older individuals. This is particularly important, as Iraq and Afghanistan-era Veterans represent a large aging cohort experiencing a high prevalence of both PTSD symptoms and pain conditions (49, 50). It is estimated that 16.5% of Veterans of these conflicts seek treatment from the Department of Veterans Affairs for co-occurring PTSD and pain (51). However, many more Veterans endorse PTSD symptoms that do not meet full criteria for a diagnosis of PTSD, as is consistent with the current findings. Specifically, the average score on the PCL-5 in this sample was 21.97, which is below the typical cut-off for probable PTSD (40). This suggests that interventions aimed at ameliorating even low levels of self-reported PTSD symptoms, particularly those above 15 on the PCL-5, may be an important area of emphasis for enhancing pain related outcomes for Veterans experiencing pain conditions.

Study analyses also contributed null findings. First, there were no significant gender differences in pain intensity or PTSD symptoms. This is consistent with previous work in Iraq and Afghanistan Veterans (52), but extends these findings to a larger age range. Second, results of exploratory analyses examining the interaction between age and PTSD symptoms on pain interference were not significant. Instead, the effect of age on pain interference was accounted for by PTSD symptoms. Although this may be surprising because pain intensity and pain interference are derived from the same measure (BPI), the result is consistent with the PCL-5 and BPI pain interference both measuring levels of burden.

Consistent with the biopsychosocial model of pain, successful reduction of PTSD symptoms may represent an effective intervention to decrease pain intensity. Currently, psychological interventions for pain tend to focus on Cognitive Behavioral Therapy or Acceptance and Commitment Therapy. These non-pharmacological approaches produce statistically significant, though small, reductions in pain intensity, but require further research due to methodological limitations, such as small sample size (53, 54). In addition to pain-focused psychotherapies, the results of the present study suggest that therapeutic approaches typically used for treatment of PTSD, such as Prolonged Exposure, Cognitive Processing Therapy for PTSD, or integrative treatments (55), may also be effective for Veterans with comorbid PTSD and pain.

There is a strong association between chronic pain and suicidality (10, 56–58) with highest risk of suicide among older white males (59). The current results suggest that treatment of PTSD symptoms, even significantly low symptoms, may be an important intervention for reducing suicidality, especially in older Veterans. Interventions aimed at reducing PTSD symptoms have been shown to be effective for reducing suicidal ideation (60), however this has not been evaluated in a pain population. Addressing pain through PTSD interventions could represent an effective intervention to reduce the risk of suicide among Veterans with comorbid pain and PTSD.

There are a number of research investigations that evaluated mechanisms underlying the relationship between PTSD symptoms and pain (61). Catastrophic thinking and avoidance are two cognitive-behavioral mechanisms that have gained significant attention as factors for maintaining both chronic pain and PTSD symptoms (61–63). However, these models have not included age. Though the effect size was relatively small, the current results suggest that age is an additional factor to consider in this relationship. It may be important to consider how avoidance and catastrophic thinking change across the lifespan, and how the subsequent changes to these cognitive-behavioral factors may affect outcomes.

The current results underscore the need for better understanding of mental health, particularly subthreshold PTSD symptoms, across the lifespan. Older adults are underrepresented in mental health research, therefore current policies may not adequately represent their interests. For example, PTSD is assumed to dissipate with age (17). However, this phenomenon could be due to changes in trauma reactions associated with aging rather than true recovery (64, 65). Further, older adults are, historically, less likely to receive treatment for PTSD (60). There is a growing recognition that mental health, and prior traumatic experiences specifically, remain a concern with advancing age (3). Additional research is necessary to better understand the mental health concerns of older adults. Future research should also inform training in geriatrics and best practices for working with Veterans at various points across their lifespan. Connecting older Veterans to treatment for PTSD symptoms and pain will remain critically important in the future, given the high prevalence of pain and mental health concerns among the Iraq and Afghanistan cohorts.

### Limitations and strengths

The data utilized for this study may not be nationally representative of the broader Veteran population, as participants primarily resided in the southeastern United States and served during the Iraq and Afghanistan war era. The age range is limited, and lacks sufficient representation at younger (e.g., under 30) and significantly older age ranges (e.g., 70+), which is congruent with Veteran characteristics from this war era. However, 18.9% of the present sample was between the ages of 60–78 and 16.4% between the ages of 30–39, which allowed for meaningful conclusions about age. All data for the present analyses were cross-sectional and cannot infer causality. Pain was presented as the outcome for the purpose of this paper, however the model variables are interchangeable in analyses due to the cross-sectional nature of the data. Thus, it is reasonable to expect that treatment of pain may have an effect on PTSD symptoms. Future studies should account for factors related to increasing age that may influence symptoms of distress (e.g., retirement from the workforce). History of mental health treatment was not investigated as a factor in the relationship between pain, PTSD symptoms, and age, which may be a key factor of interest in future longitudinal work. Using a standalone assessment of symptom validity is a strength of this study.

## Conclusion

The results of this study demonstrate that the interaction between age and greater PTSD symptoms may exacerbate pain severity among Veterans. Whereas previous research has demonstrated a positive association between pain and age and a negative association between age and PTSD symptoms, addressing the interaction effects of these factors adds a new dimension to the current understanding of both pain and PTSD in aging Veterans. These preliminary findings suggest that treatment of PTSD symptoms may provide an additional avenue by which to address pain, especially among older cohorts.

## Clinical impact

This study contributes additional understanding of the relationship between pain and PTSD across the lifespan. When greater PTSD symptoms are present at older ages, Veterans report greater pain intensity. Providers should assess for trauma and PTSD symptoms among older Veterans reporting pain as a potential additional avenue of treatment.

## Author’s note

VO’C, PhD, Research & Academic Affairs Service Line, W. G. (Bill) Hefner VA Healthcare System, Salisbury, NC, United States; Veterans Integrated Service Networks (VISN)-6 Mid-Atlantic Mental Illness, Research Education and Clinical Center (MIRECC), Durham, NC, United States; Department of Neurology, Wake Forest School of Medicine, Winston-Salem, NC, United States. JR, PhD, Research & Academic Affairs Service Line, W. G. (Bill) Hefner VA Healthcare System, Salisbury, NC; VISN-6 Mid-Atlantic MIRECC, Durham, NC, United States; Department of Neurobiology & Anatomy, Wake Forest School of Medicine, Winston-Salem, NC, United States. JN, PhD, VISN-6 Mid-Atlantic MIRECC, Durham, NC, United States; Durham Veterans Affairs Health Care System, Durham, NC, United States; Department of Psychiatry and Behavioral Sciences, Duke University School of Medicine, Durham, NC, United States. AM, PsyD, MA-MIRECC, Research & Academic Affairs Service Line, W. G. (Bill) Hefner VA Healthcare System, Salisbury, NC, United States; Department of Neurology, Wake Forest School of Medicine, Winston-Salem, NC, United States. KC, BFA, MA-MIRECC, Research & Academic Affairs Service Line, W. G. (Bill) Hefner VA Healthcare System, Salisbury, NC, United States. HM, PhD, Mental Health & Behavioral Sciences Service Line, W. G. (Bill) Hefner VA Healthcare System, Salisbury, NC, United States; Department of Neurology, Wake Forest School of Medicine, Winston-Salem, NC, United States. SM, PhD, MA-MIRECC, Research & Academic Affairs Service Line, W. G. (Bill) Hefner VA Healthcare System, Salisbury, NC, United States; Department of Physiology & Pharmacology, Wake Forest School of Medicine, Winston-Salem, NC, United States.

## Data availability statement

The original contributions presented in the study are included in the article/supplementary material, further inquiries can be directed to the corresponding author.

## Ethics statement

The studies involving human participants were reviewed and approved by Hefner VAHCS Institutional Review Board. The patients/participants provided their written informed consent to participate in this study.

## Author contributions

VO’C: conceptualization, formal analysis, investigation, methodology, visualization, writing – original draft, writing – review and editing. JR: conceptualization, investigation, project administration, supervision, writing – review and editing. JN: conceptualization, writing – review and editing. AM and HM: investigation, writing – review and editing. KC: investigation, writing – review and editing. VA Mid-Atlantic MIRECC Workgroup: data curation, funding acquisition, project administration, resources. SM: conceptualization, data curation, formal analysis, investigation, methodology, supervision, visualization, writing – original draft, writing – review and editing. All authors contributed to the article and approved the submitted version.

## VA Mid-Atlantic MIRECC Workgroup

The Mid-Atlantic MIRECC Workgroup contributors for this paper include: Jean C. Beckham, PhD, Patrick S. Calhoun, PhD, Eric Dedert, PhD, Eric B. Elbogen, PhD, John A. Fairbank, PhD, Robin A. Hurley, MD, Jason D. Kilts, PhD, Nathan A. Kimbrel, PhD, Angela Kirby, MS, Scott D. McDonald, PhD, Christine E. Marx, MD, MS, Scott D. Moore, MD, PhD, Rajendra A. Morey, MD, Robert D. Shura, PsyD, Cindy Swinkels, PhD, Larry A. Tupler, PhD, Elizabeth E. Van Voorhees, PhD, Tate F. Halverson, Ph.D., Pallavi Aurora, PhD., and Brandy S. Martinez, PhD.

## Funding

This work was supported by the Salisbury VA Health Care System, VA Mid-Atlantic (VISN 6) Mental Illness Research, Education, and Clinical Center (MIRECC), and the Department of Veterans Affairs Office of Academic Affiliations Advanced Program in Mental Illness, Research, and Treatment.

## Conflict of interest

The authors declare that the research was conducted in the absence of any commercial or financial relationships that could be construed as a potential conflict of interest.

## Publisher’s note

All claims expressed in this article are solely those of the authors and do not necessarily represent those of their affiliated organizations, or those of the publisher, the editors and the reviewers. Any product that may be evaluated in this article, or claim that may be made by its manufacturer, is not guaranteed or endorsed by the publisher.

## Author disclaimer

The views, opinions and/or findings contained in this article are those of the authors and should not be construed as an official Veterans Affairs position, policy, or decision, unless so designated by other official documentation.
